# The efficacy and safety of thrombolytic agents for patients with cerebral infarction: A protocol for systematic review and network meta-analysis

**DOI:** 10.1097/MD.0000000000013762

**Published:** 2018-12-28

**Authors:** Shenghui Huang, Junshi Jiang, Zhiming Xu, Cuncun Lu, Fang Cao, Wengxia Sang, Ting Gong, Yanyi Li

**Affiliations:** a Department of Neurology, Affiliated Hospital of Gansu University of Chinese Medicine; b College of Integrated Traditional Chinese and Western Medicine, Guansu University of Chinese Medicine; c Evidence-based Medicine Center, School of Basic Medical Sciences, Lanzhou University; d Department of Encephalopathy, Gansu Provincial Hospital of Traditional Chinese Medicine, Lanzhou, China.

**Keywords:** cerebral infarction, network meta-analysis, systematic review, thrombolytic agent

## Abstract

**Background:**

Cerebral infarction is a commonly dangerous disease also with high morbidity and mortality. Thrombolytic agent is an effective method to treat it, but their relative efficacy and safety are unclear. A network meta-analysis (NMA) will be conducted to resolve this urgent problem.

**Methods:**

The PubMed, Embase, and Cochrane library will be systematically search from their inception to November 2018. All randomized controlled trials (RCTs) will be included this NMA and their risk of bias will be assessed using Cochrane handbook tool. The outcomes of efficacy and safety including: Modified Rankin Scale scores, reperfusion rate, incidence of symptomatic intracerebral hemorrhage and all-cause mortality. A network meta-analysis will be performed using R x64 3.5.1 software and pairwise meta-analysis will be conducted using Stata 12.0 software (Stata Corp., College Station, Texas). Grading of Recommendations Assessment, Development and Evaluation (GRADE) will be used to assess quality of outcomes.

**Results:**

The results of NMA will be published in a peer-reviewed journal.

**Conclusion:**

The NMA will provide a comprehensive evidence summary on thrombolytic agents for patients with cerebral infarction.

## 1 Introduction

Cerebral infarction is a commonly dangerous disease also with high morbidity and mortality.^[1]^ It is estimated that about 5.5 million people die from cerebral infarction annually around the world,^[2]^ because most of people no receive effective and timely treatment.^[3]^ As is known to all, thrombolytic therapy is an effective way to treat cerebral infarction, but, there are so many thrombolytic agents,^[4]^ for example, urokinase, streptokinase, alteplase, etc. And to the best of our knowledge, there is no a study to compare their efficacy and safety, so it is a big obstacle for clinicians to prescribe them reasonably.

Network meta-analysis (NMA) can simultaneously compare multiple interventions for a specific problem and improve the statistic power.^[5]^ And now, it has been popular with researchers and clinicians to assess the relative effectiveness among multiple interventions.^[6]^ Eventually, it will provide a result of comprehensive ranking to reference for clinical practice.^[7]^ Therefore, we will conduct an NMA to compare the efficacy and safety of thrombolytic agents for patients with cerebral infarction in adherence to Cochrane handbook^[8]^ strictly.

## 2 Methods

### 2.1 Study registration

This study protocol has been registered on PROSPERO: CRD42018110419.

### 2.2 Eligibility criteria

#### 2.2.1 Type of study

Randomized controlled trials (RCTs) that compared the effect of different thrombolytic agents for patients with cerebral infarction will be included in this NMA. And no any limitation will be used for the study.

#### 2.2.2 Participants

We will include patients (aged 18 years or older) with cerebral infarction, who scheduled for thrombolytic therapy with thrombolytic agents. And not limited to race, area, sex, etc.

#### 2.2.3 Interventions

All RCTs reported different thrombolytic agents for patients with cerebral infarction will be included. And not limited to doses, frequency, and manufacturer, etc.

### 2.3 Outcomes

The efficacy outcomes including: Modified Rankin Scale scores (mRSs), reperfusion rate. The safety outcomes including: incidence of symptomatic intracerebral hemorrhage (sICH), all-cause mortality. Randomized controlled trials reporting above at least one outcome will be included the present study.

### 2.4 Data source

The PubMed, Embase, and Cochrane library will be systematically search using Mesh and key words from their inception to November 2018. The searching strategy of PubMed was as follows:

#1 “Thrombolytic Therapy”[Mesh] OR “Fibrinolytic Agents”[Mesh]

#2 “thrombolytic therapy”[Title/Abstract] OR “thrombolytic therapies”[Title/Abstract] OR thrombolysis[Title/Abstract] OR thrombolyses[Title/Abstract] OR “fibrinolytic therapy”[Title/Abstract] OR “fibrinolytic therapies”[Title/Abstract] OR “fibrinolytic agent”[Title/Abstract] OR “fibrinolytic agents”[Title/Abstract] OR “fibrinolytic drug”[Title/Abstract] OR “fibrinolytic drugs”[Title/Abstract] OR “thrombolytic agent”[Title/Abstract] OR “thrombolytic agents”[Title/Abstract] OR “thrombolytic drug”[Title/Abstract] OR “thrombolytic drugs”[Title/Abstract] OR “antithrombotic agent”[Title/Abstract] OR “antithrombotic agents”[Title/Abstract] OR “antithrombic drug”[Title/Abstract] OR “antithrombic drugs”[Title/Abstract] OR urokinase[Title/Abstract] OR streptokinase[Title/Abstract] OR “tissue plasminogen activator”[Title/Abstract] OR “recombinant tissue plasminogen activator”[Title/Abstract] OR reteplase[Title/Abstract] OR deaminase[Title/Abstract] OR tenecteplase[Title/Abstract]

#3 #1 OR #2

#4 “Stroke”[Mesh]

#5 stoke[Title/Abstract] OR stokes[Title/Abstract] OR “cerebrovascular accident”[Title/Abstract] OR “cerebrovascular accidents”[Title/Abstract] OR “CVA”[Title/Abstract] OR “CVAs”[Title/Abstract] OR “brain vascular accident”[Title/Abstract] OR “brain vascular accidents”[Title/Abstract] OR apoplexy[Title/Abstract] OR apoplexia[Title/Abstract] OR apoplexies[Title/Abstract] OR “cerebral infarction”[Title/Abstract] OR “cerebral infarctions”[Title/Abstract] OR “brain infarction”[Title/Abstract] OR “brain infarctions”[Title/Abstract]

#6 #4 OR #5

#7 “Randomized Controlled Trial” [Publication Type] OR “Randomized Controlled Trials as Topic”[Mesh] OR “Controlled Clinical Trial” [Publication Type]

#8 random∗[Title/Abstract]

#9 #7 OR #8

#10 #3 AND #6 AND #9

### 2.5 Study selection

The Endnote X7 which is a literature management software will be used to manage records from databases. Screening process will include 2 stages, first, two experienced reviewers will independently check the title and abstract of all records using Endnote X7 to find appropriate studies according to our eligibility criteria; second, each full text from first stage will be downloaded and to further check. Any disagreement will be resolved through discussion. To extract relevant information, a detailed extraction form will be created using Microsoft Excel 2016, and mainly information including: first author, year of study, sample size, patient characteristics, interventions, therapeutic regimen and doses, and outcomes. The third author will examine all extracted information to decrease bias. When data were reported as median rather than mean, and range or interquartile rather than standard deviation, the mean and standard deviation will be estimated using method from Hozo et al.^[9]^

### 2.6 Risk of bias (ROB) assessment

Two reviewers will independently assess the risk of bias for all included studies using the Cochrane handbook tool.^[8]^ And this tool including 6 domains: random sequence generation, allocation concealment, blind, incomplete outcome data, selective reporting, and other bias. The process also will be implemented with 2 reviewers independently and difference through discussion to reach agreement.

### 2.7 Statistical analysis

#### 2.7.1 Pairwise meta-analyses

The Stata 12.0 software will be used to perform pairwise meta-analyses with random-effects model. Dichotomous outcomes will be measured using relative risk (RR) with 95% confidence interval (95% CI), and the mean difference (MD) with 95%CI will be presented for continuous outcomes. The potential heterogeneity will be measured using *I*^2^, when the *I*^2^ >50% and *P* < .1, subgroup analysis will be performed to explore the heterogeneity. Publication bias will be tested using Begg and Egger funnel plot^[10,11]^ when the number of included studies no <10.^[12]^

#### 2.7.2 Network meta-analyses

The R x64 3.5.1 software will be used to performed a Bayesian NMA, also, dichotomous data will be reported as relative risk (RR) with 95% confidence interval (95% CI), and the mean difference (MD) with 95%CI will be reported for continuous data. The inconsistency between direct and indirect comparisons will be tested using node splitting method.^[13]^ Surface under the cumulative ranking area (SUCRA) will be used to rank the different thrombolytic agents. Network geometry will use nodes to represent different agents and edges to represent the head to-head agents. And the size of node represents sample sizes of intervention, thickness of edge represents numbers of included studies.

### 2.8 Quality of evidence node

The quality of evidence for all outcomes will be assess using the Grading of Recommendations Assessment, Development and Evaluation (GRADE),^[14]^ mainly considerations including: risk of bias, inaccuracy, inconsistency, indirectness, publication bias, and results of assessment will be graded 4 levels: very low, low, moderate, and high level.

## 3 Discussion

Now, many thrombolytic agents for cerebral infarction are exists, but there is no confirmed evidence on their efficacy and safety. Therefore, we will conduct an NMA to fill this gap according to Cochrane handbook and the PRISMA extension statement^[15]^ for NMAs. The study will provide a comprehensive evidence of different thrombolytic agents for patients with cerebral infarction and we hope the result will provide reference for cerebral infarction treatment. And the present protocol is designed in adherence to PRISMA-P^[16]^ which is used to reporting protocol of systematic review.

## Author contributions

**Data curation:** Shenghui Huang, Junshi Jiang, Zhiming Xu, Cuncun Lu.

**Methodology:** Shenghui Huang, Cuncun Lu, Fang Cao, Ting Gong.

**Resources:** Wengxia Sang.

**Software:** Shenghui Huang, Junshi Jiang.

**Writing – original draft:** Shenghui Huang, Yanyi Li.

**Writing – review & editing:** Shenghui Huang, Yanyi Li.
